# Transient monocular blindness and the risk of vascular complications according to subtype: a prospective cohort study

**DOI:** 10.1007/s00415-016-8189-x

**Published:** 2016-06-17

**Authors:** Eline J. Volkers, Richard C. J. M. Donders, Peter J. Koudstaal, Jan van Gijn, Ale Algra, L. Jaap Kappelle

**Affiliations:** 1 University Department of Neurology and Neurosurgery, Brain Center Rudolf Magnus, University Medical Center Utrecht, Utrecht, The Netherlands; 2 Julius Center for Health Sciences and Primary Care, University Medical Center Utrecht, Utrecht, The Netherlands; 3 Department of Neurology, Diakonessenhuis, Utrecht, The Netherlands; 4 Department of Neurology, Erasmus Medical Center, Rotterdam, The Netherlands

**Keywords:** Amaurosis fugax, History symptoms, Transient ischemic attack, Etiology

## Abstract

Patients with transient monocular blindness (TMB) can present with many different symptoms, and diagnosis is usually based on the history alone. In this study, we assessed the risk of vascular complications according to different characteristics of TMB. We prospectively studied 341 consecutive patients with TMB. All patients were interviewed by a single investigator with a standardized questionnaire; reported symptoms were classified into predefined categories. We performed Cox regression analyses with adjustment for baseline vascular risk factors. During a mean follow-up of 4.0 years, the primary outcome event of vascular death, stroke, myocardial infarction, or retinal infarction occurred in 60 patients (annual incidence 4.4 %, 95 % confidence interval (CI) 3.4–5.7). An ipsilateral ischemic stroke occurred in 14 patients; an ipsilateral retinal infarct in six. Characteristics of TMB independently associated with subsequent vascular events were: involvement of only the peripheral part of the visual field (hazard ratio (HR) 6.5, 95 % CI 3.0–14.1), constricting onset of loss of vision (HR 3.5, 95 % CI 1.0–12.1), downward onset of loss of vision (HR 1.9, 95 % CI 1.0–3.5), upward resolution of loss of vision (HR 2.0, 95 % CI 1.0–4.0), and the occurrence of more than three attacks (HR 1.7, 95 % CI 1.0–2.9). We could not identify characteristics of TMB that predicted a low risk of vascular complications. In conclusion, careful recording the features of the attack in patients with TMB can provide important information about the risk of future vascular events.

**Electronic supplementary material:**

The online version of this article (doi:10.1007/s00415-016-8189-x) contains supplementary material, which is available to authorized users.

## Introduction

Transient monocular blindness (TMB) or amaurosis fugax [1] is usually attributed to temporary ischemia of the retina, or part of it. Therefore, these episodes are considered transient ischemic attacks (TIAs) in the territory of the ipsilateral internal carotid artery (ICA) [2]. In general, patients with TMB associated with atheromatous disease in the carotid artery have an average 1 year risk of recurrent stroke of 2 % [3, 4], but in patients with severe ICA stenosis the risk of ipsilateral stroke is up to 16 % after 3 years [5]. Overall, the risk is two to three times lower than in patients with cerebral TIA [5, 6].

The diagnosis of TMB may be difficult, because the history is the only source of information while the range of visual symptoms is almost unlimited [7, 8]. Consequently, management can be difficult as well; because it is unknown which patterns are most relevant with respect to future vascular complications. Therefore, it is often challenging to distinguish between TMB with a high risk versus low risk.

Earlier research on the predictive value of history characteristics of TMB focused on the relationship between visual symptoms and presence of ipsilateral carotid atherosclerotic disease or other sources of emboli [8–10].

For the current study, we hypothesized that specific patterns of transient monocular loss of vision may carry a different prognosis, which might guide clinicians in differentiating between serious and more benign causes of TMB.

## Methods

### Patients

Between September 1992 and March 1996, we prospectively included 341 consecutive patients from 18 centers. Patients were required to have experienced sudden, transient loss of vision in one eye in the previous 6 months; the attacks did not last longer than 24 h and were not caused by known ophthalmological disease.

This study was approved by the central research ethics committee of the University Medical Center Utrecht, The Netherlands; all patients gave written informed consent to participate.

### History characteristics

All patients were interviewed about the details of the attack by one and the same investigator (RCJMD). The median interval between the last attack and the interview was 8 weeks. This detailed history was guided by a standardized questionnaire (Supplementary Table 1). If the patient did not spontaneously mention a characteristic of a given category, the possibilities in that category were read out by the investigator, after which the patient could answer ‘yes’, ‘no’, or ‘I do not know’. If a symptom not listed in Supplementary Table 1 was mentioned by the patient, the characteristic was recorded in the patient’s own words; afterwards two of us (RCJMD, LJK) classified them as belonging to one of the predefined categories, without having access to the results of ancillary investigations. Disagreements on this classification were resolved by discussion. To exclude relevant ophthalmological disease we referred all patients for consultation to an ophthalmologist.

**Table 1:** Baseline characteristics

Variable	Total study population (*n* = 341)
Age, years (mean, SD)	61.9 (12.3)
Male sex	196 (57 %)
History of	
Diabetes mellitus	22 (7 %)
Hypertension	150 (44 %)
Intermittent claudication	67 (20 %)
Myocardial infarction	52 (15 %)
Atrial fibrillation	12 (4 %)
Angina pectoris	78 (23 %)
TIA or stroke	106 (31 %)
Migraine^a^	42 (13 %)
Smoking	127 (37 %)
Total cholesterol ≥6.0 mmol/L	199 (58 %)
Medication use at baseline	
Antithrombotic	
Antiplatelet	231 (68 %)
Anticoagulant	60 (18 %)
Both antiplatelet and anticoagulant	14 (4 %)
Antihypertensive	110 (32 %)
Lipid-lowering	22 (7 %)
Antidiabetic	10 (3 %)
Degree of stenosis of ipsilateral ICA^a, b^	
No stenosis	158 (47 %)
Mild or moderate stenosis (1–69 %)	33 (10 %)
Severe stenosis (≥70–99 %)	100 (30 %)
Occlusion	45 (13 %)
CEA performed on ipsilateral side during study	81 (24 %)
Delay between first event and interview, days (median, IQR)	56 (17.5–107.0)

^a^Unknown in five patients

^b^According to duplex ultrasonography performed at baseline

*SD* standard deviation, *TIA* transient ischemic attack, *ICA* internal carotid artery, *CEA* carotid endarterectomy, *IQR* interquartile range

### Baseline characteristics

The following patient characteristics were recorded: age, sex, current smoking, diabetes mellitus, hypercholesterolemia, atrial fibrillation, coronary artery disease (history of angina or myocardial infarction), hypertension, intermittent claudication, previous TIA or stroke, migraine, or glaucoma. The overall handicap of patients was graded with the modified Rankin scale [11, 12]. Blood tests were performed for total cholesterol and an electrocardiogram was obtained. Patients underwent duplex ultrasonography of the carotid arteries at study entry. Atherosclerotic lesions of the ipsilateral ICA were graded according to peak systolic velocities on duplex ultrasonography and categorized into the following categories: normal diameter, stenosis between 1 and 69 %, stenosis between 70 and 99 %, and occlusion.

### Follow-up

We followed all patients for a period of at least 3 years, with six monthly intervals. The follow-up contact consisted of a hospital visit, or it was performed through a standardized telephone interview by the study coordinator. Each time we ascertained the occurrence of possible outcome events, the continuation of the attacks, or other manifestations of vascular disease.

### Outcome events

The primary outcome event was the composite of death from all vascular causes, non-fatal stroke, non-fatal myocardial infarction, or retinal infarction, whichever happened first. The secondary outcome event was the occurrence of ipsilateral ischemic stroke or ipsilateral retinal infarction.

The definition of death from vascular causes included sudden death, cardiac death, or death from stroke. For the diagnosis of non-fatal stroke, sudden and focal neurological deficits persisting for more than 24 h had to correspond with a new infarct or hemorrhage on a repeated CT scan, or had to cause a permanent increase in handicap of at least one grade on the modified Rankin scale [11, 12]. Myocardial infarction had to be documented by at least two of the following characteristics: a history of chest discomfort, specific cardiac enzyme levels more than twice the upper limit of normal, or development of Q waves on the standard 12-lead electrocardiogram. Retinal infarction had to be documented by a history of persistent loss of vision in one eye and by typical changes on funduscopy. All outcome events were independently classified by three members of the auditing committee for outcome events. The committee members were unaware of the symptoms reported by the patient at baseline and the results from ancillary investigations.

### Statistical analysis

We estimated that during a 3 years inclusion period 210 patients could be entered into the study and that each patient could be followed for at least 3 years. Thus, 945 patient follow-up years could be obtained. In the Dutch TIA trial, conducted at the end of the 1980s, the rate of vascular events of patients with TMB was 3 % per year (Dutch TIA trial; unpublished data). On the assumption a history characteristic has a prevalence of 50 %, a risk ratio of 3 would have a 95 % confidence interval (CI) of 1.3–7.0.

We related features of TMB with the occurrence of outcome events in terms of hazard ratios (HRs), with corresponding 95 % CIs by means of Cox proportional hazards modeling. For those features of TMB that showed a statistically significant association with outcome in univariable analysis, we additionally performed adjusted analyses. In consecutive bivariable analyses we assessed which other patient characteristics including age, sex, and presence of vascular risk factors changed the crude hazard ratio importantly. These characteristics were taken into account in multivariable analysis to obtain fully adjusted estimates. The maximum number of characteristics that could be adjusted for was limited by the total number of outcome events; one variable for every ten outcome events.

We made Kaplan–Meier curves to graphically assess the cumulative risks of the features of the history that showed a statistically significant relationship with the primary outcome event.

## Results

The mean age of the 341 patients was 61.9 ± 12.3 years; 57 % were male (Table 1). Mean follow-up was 4.0 years (median 4.1), resulting in 1364 patient years. Two patients were lost to follow-up after event-free periods of 3.7 and 1.7 years, respectively. Ophthalmological examination was performed in 299 (88 %) patients; in the other 42 patients it was not possible for logistic reasons. Funduscopy of the symptomatic eye showed permanent arterial branch occlusion in four patients and possible symptomatic retinal emboli in eight patients; the emboli were judged to consist of cholesterol in six patients and of platelets or calcified material in two other patients. A chronic ischemic syndrome of the eye [13] was diagnosed in five patients. Increased ocular pressure was found to coexist in three patients, one of whom had a concurrent ischemic syndrome of the eye.

Baseline characteristics are summarized in Table 1. In 296 (87 %) patients at least one of the following vascular risk factors was present: diabetes mellitus, hypertension, smoking, or total cholesterol of ≥6.0 mmol/L. The proportion of patients who used antithrombotic medication at baseline was comparable with the proportion of patients who used antithrombotic medication during follow-up.

The primary (composite) outcome event occurred in 60 patients (annual incidence 4.4 %, 95 % CI 3.4–5.7 %). Ipsilateral ischemic stroke occurred in 14 patients (1.0 %/year) and ipsilateral retinal infarction in six patients (0.4 %/year). Myocardial infarction occurred in 20 patients (1.5 %/year). Forty-six (13 %) patients died, 22 (6 %) from a vascular cause. Of these 22, three patients died from ischemic stroke, three from hemorrhagic stroke, and 16 from other vascular causes.

**Fig. 1:**
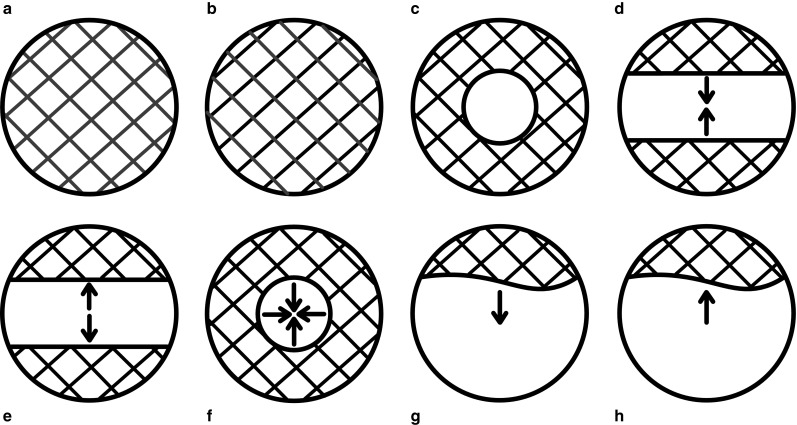
Schematic illustrations of different visual field loss patterns. **a** Completely colored visual field, **b** completely *black* visual field with color, **c** involvement of peripheral visual field only, **d** onset of curtain to above or below, **e** resolution of curtain to above or below, **f** constricting onset of visual field loss, **g** downward onset of loss of vision, **h** upward resolution of loss of vision

Table 2 and Supplementary Fig. 1 show the features of the history that had a statistically significant association on univariable analysis with the occurrence of the primary outcome measure. In Fig. 1 the different patterns of visual field loss are illustrated. On bivariable analysis, presence of the following characteristics influenced HRs of the nine features of TMB the most: sex, history of intermittent claudication, history of myocardial infarction, history of angina pectoris, and severe stenosis or occlusion of ipsilateral ICA. After adjustment for these factors, involvement of the peripheral but not the central part of the visual field, constricting onset of visual field loss, downward onset of loss of vision, upward resolution of loss of vision, and occurrence of more than three attacks remained statistically significant predictors of the outcome (Table 2). We could not identify a feature of the history that was consistently associated with a good outcome with respect to the primary outcome event.

**Table 2:** Occurrence of primary outcome (composite of vascular death, stroke, myocardial infarction, or retinal infarction) during follow-up

History characteristic	Risk of primary outcome			
	Characteristic present *n* (%)	Characteristic absent *n* (%)	Crude HR with 95 % CI	Adjusted HR with 95 % CI^a^
Completely colored VF	5/12 (42 %)	55/329 (17 %)	2.9, 1.2–7.3	2.0, 0.8–5.2
Both black and colored VF	3/6 (50 %)	57/335 (17 %)	3.4, 1.0–10.7	3.0, 0.9–10.1
Peripheral VF only	8/11 (73 %)	52/330 (16 %)	7.2, 3.4–15.2	6.5, 3.0–14.1
Altitudinal onset	16/57 (28 %)	44/284 (16 %)	2.0, 1.1–3.5	1.4, 0.8–2.5
Altitudinal resolution	15/55 (27 %)	45/286 (16 %)	1.8, 1.0–3.3	1.5, 0.8–2.7
Constricting onset	3/8 (38 %)	57/333 (17 %)	3.1, 1.0–9.8	3.5, 1.0–12.1
Downward onset	14/41 (34 %)	46/300 (15 %)	2.5, 1.4–4.6	1.9, 1.0–3.5
Upward resolution	11/33 (33 %)	49/308 (16 %)	2.4, 1.3–4.7	2.0, 1.0–4.0
Occurrence of >3 attacks	34/139 (25 %)	26/202 (13 %)	2.1, 1.2–3.5	1.7, 1.0–2.9

^a^Adjusted for: sex, intermittent claudication, myocardial infarction, angina pectoris, severe stenosis (≥70–100 %) or occlusion of ipsilateral ICA

*VF* visual field, *HR* hazard ratio, *CI* confidence interval, *ICA* internal carotid artery

Features of TMB that predicted the secondary outcome event on univariable analysis are summarized in Table 3. Presence of ipsilateral ICA stenosis of 70 % or more or occlusion had the largest influence on the crude HRs. All risk estimates remained essentially the same after adjustment for this factor (Table 3). Adjustment for carotid endarterectomy (CEA) performed on the ipsilateral ICA during the study did not alter the HRs for both the primary and secondary outcome measures. Also, adjustment for the interval between last event and CEA hardly altered the risk of primary or secondary outcome events in the 81 patients who underwent ipsilateral CEA during the study (data not shown).

**Table 3:** Occurrence of secondary outcome (ipsilateral (non-)fatal ischemic stroke or retinal infarction) during follow-up

History characteristic	Risk of secondary outcome			
	Characteristic present *n* (%)	Characteristic absent *n* (%)	Crude HR with 95 % CI	Adjusted HR with 95 % CI^a^
Completely colored VF	3/12 (25 %)	17/329 (5 %)	5.3, 1.5–18.1	5.7, 1.6–19.6
Both black and colored VF	2/6 (33 %)	18/335 (5 %)	7.1, 1.6–30.8	6.9, 1.6–30.2
Peripheral VF only	4/11 (36 %)	16/330 (5 %)	8.3, 2.8–24.9	5.4, 1.8–16.5
Altitudinal onset	8/57 (14 %)	12/284 (4 %)	3.5, 1.4–8.6	2.5, 1.0–6.2
Altitudinal resolution	9/55 (16 %)	11/286 (4 %)	4.4, 1.8–10.7	3.8, 1.6–9.2
Downward onset	6/41 (15 %)	14/300 (5 %)	3.3, 1.3–8.7	2.6, 1.0–6.9
Upward resolution	6/33 (18 %)	14/308 (5 %)	4.3, 1.6–11.2	4.1, 1.6–10.7

^a^Adjusted for: severe stenosis (≥70–100 %) or occlusion of ipsilateral ICA

*VF* visual field, *HR* hazard ratio, *CI* confidence interval, *ICA* internal carotid artery

## Discussion

This is the largest prospective series of patients with TMB studied to date, and the only study that relates history characteristics of TMB with clinical outcome events during follow-up. We found that the risk of serious vascular events in patients with TMB varies according to certain elements of the history, independently from baseline vascular risk factors.

Clinical features of TMB caused by emboli [2] may resemble those caused by related diseases, such as (retinal) migraine [14–16], anterior ischemic optic neuropathy, or ophthalmological disorders. The usual assumption is that positive phenomena often represent migrainous attacks [15]. In the present study, a history of migraine was indeed associated with a good outcome (data not shown). However, since a history of migraine had little influence on the HRs of the features of TMB on bivariable analysis, we did not adjust for this factor.

We could not confirm the benign nature of positive phenomena in this study, in agreement with two other studies [10, 17]. Moreover, complete uniform coloring of the visual field was found to be a harbinger of cardiovascular complications in our study. Although the association with the primary (composite) outcome was non-significant after adjustment, completely colored visual field remained a significant predictor of the secondary outcome (Tables 2 and 3). This difference could be explained by the inclusion of vascular death and myocardial infarction in the primary outcome measure, which might have diluted the association between history characteristics and the occurrence of stroke and retinal infarction.

Three history characteristics that predicted serious vascular events (downward onset of loss of vision, upward resolution of loss of vision, and occurrence of more than three attacks) fit the classical notions about TMB best [18]. Attacks with a downward onset of visual field loss were attributed to emboli in an earlier study [8]. Upward resolution of visual field loss might occur when restriction of blood flow to the lower part of the retina is restored last. In other cohorts of patients with TMB or TIA in general, the occurrence of multiple attacks has also been found to predict recurrent vascular events [4, 6]. Involvement of the peripheral, but not the central area of the visual field was identified as the most powerful predictor of impending vascular disease in our study. In this group of patients, 1 out of 11 had a history of migraine, whereas 8 patients had an ipsilateral stenosis or occlusion of the ICA, thereby having a higher risk of stroke. This is contrary to the results of our previous retrospective study, in which partial involvement of the visual field carried a significantly lower risk of the composite vascular outcome compared with complete blindness of one eye [19]. These conflicting results can be explained by the lack of standardization in the collection of data on symptoms of TMB in our previous study, which might have induced information bias. The fifth history characteristic that predicted vascular events in our study—a constricting onset—was associated with migraine in earlier studies [20, 21], and with a lower risk of carotid atheromatous disease or a cardiac source of emboli [8]. However, in our study, 3/7 patients reporting a constricting onset had a severe stenosis or occlusion of the ipsilateral ICA, which again raises stroke risk.

Three earlier studies distinguished subtypes of TMB according to presumed pathophysiology [8–10]. However, none of these studies addressed the prognostic implications of the different patterns. It is intriguing that neither the present study nor other studies could identify a subtype of TMB that can confidently be characterized as ‘innocuous’, which might have helped to explain that after ocular attacks the overall risk of major vascular events is two to three times lower than after hemispheral attacks [5, 22].

The incidence, prognosis and treatment of TMB in general have been extensively studied in both community and hospital based studies [3, 4, 6, 23–26]. In our cohort, the overall annual incidence of cardiovascular complications was 4.4 % (95 % CI 3.4–5.7 %), which is somewhat higher than in more recent studies [4]. As in other studies, the incidence of retinal infarctions was low [19, 23, 25, 27]. Overall, patients with TMB associated with atheromatous disease have a lower risk of ipsilateral stroke than patients with cerebral TIA [5, 6]. In contrast, we found that the risk of cardiac disease in patients with TMB was not lower than that reported in studies on cerebral TIA [23, 28]. In the present cohort of patients with TMB, atypical features of the attack did not carry a higher risk of cardiac events (data not shown), which was confirmed in a recent study [29].

The prevalence of ipsilateral ICA stenosis or occlusion in our cohort was much higher than in a recent prospective study of patients with TMB who were referred to a hospital TIA-clinic with 24 h access (42.5 vs. 12.5 %) [4]. However, patients in our cohort probably had more severe vascular disease and atherosclerosis, reflected by a higher prevalence of vascular risk factors and vascular comorbidity than in the TIA-clinic study. The authors of the TIA-clinic study state in their discussion that the prevalence of ICA stenosis among the included TMB patients was unexpectedly low compared with earlier studies, and argue that this lower prevalence may be explained by population differences [4].

Strengths of our study are that all patients were included according to strict criteria, with exclusion of patients with known ophthalmological disease, and that a single physician systematically interviewed all patients with a standardized questionnaire. Additionally, we adjusted for potential confounders to ensure the associations we found were independent from vascular risk factors. A limitation of our study is that the cohort is hospital based, and although it was a multicenter study, the majority of patients were referred to university hospitals with facilities for CEA. This is reflected in the relatively low age of the patients. Patients who are referred to centers with CEA facilities may more often have unequivocal TMB symptoms or have more vascular risk factors; hence, it is possible that we missed patients with relatively benign forms of TMB. Therefore, the results cannot be extrapolated to all patients with TMB. Also, recruitment took place from 1992 to 1996, which might limit generalizability of our results due to improved medical and surgical management of patients with TMB in the last decades. However, the higher rate of vascular events in our cohort facilitated the study of the relationship between history symptoms and the occurrence of the outcomes. Because of logistic reasons we did not report these results soon after they were collected; the continuing clinical problem of being confronted with a variety of transient visual symptoms prompted us to publish our results as yet. We cannot rule out that our results are influenced by chance, because we used many history characteristics in the univariable analysis. Yet, symptoms that showed a significant association with the primary and secondary outcome measures are very similar (Tables 2 and 3), which reduces the possibility of chance. Finally, the risk of recurrent stroke is highest within the first 2 weeks after a TIA [30]. Therefore, the median delay of 8 weeks between the last symptoms and entry into the study may have induced information bias because of missing outcome events and their associated history characteristics. On the other hand, all patients were interviewed with a standardized questionnaire, which limits the risk of recall bias.

In conclusion, the results of our study show that subtypes of TMB carry a relatively high risk of vascular complications. Careful history taking may help to assess the prognosis of an individual patient. In particular, involvement of the peripheral but not the central part of the visual field, a constricting onset of visual field loss, downward onset of loss of vision, upward resolution of loss of vision, and the occurrence of multiple attacks of TMB all independently carried a relatively high risk of vascular complications. We could not identify any ‘benign’ prognostic features of TMB. Therefore, the presence of unusual or otherwise atypical symptoms should not be a reason to withhold secondary prevention to reduce the risk of future vascular events.

## Electronic supplementary material

Below is the link to the electronic supplementary material.

Supplementary material 1 (PDF 113 kb)

Supplementary material 2 (PDF 118 kb)
