# Supplementary material for: Transient monocular blindness and the risk of vascular complications according to subtype: a prospective cohort study
Source: J Neurol. 2016 Jun 17;263(9):1771–7. doi: 10.1007/s00415-016-8189-x (PMC5010823; doi:10.1007/s00415-016-8189-x)
Supplement: Supplementary file 1 — Supplementary material 1 (PDF 113 kb) [file 415_2016_8189_MOESM1_ESM.pdf]

ELECTRONIC SUPPLEMENTARY MATERIAL

Article title:

Transient monocular blindness and the risk of vascular complications according to subtype: a prospective cohort study

Journal name:

Journal of Neurology

Authors:

Eline J. Volkers MD, Richard C.J.M. Donders MD, Peter J. Koudstaal MD, Jan van Gijn MD, Ale Algra MD, L. Jaap Kappelle MD

Address for correspondence:

E.J. Volkers, Julius Center for Health Sciences and Primary Care, University Medical Center Utrecht

Internal mail no. Str. 6.131, P.O. Box 85500, 3508 GA Utrecht, The Netherlands

Phone: +31.88.7569626

Fax: +31.88.7568099

Email: E.J.Volkers@umcutrecht.nl

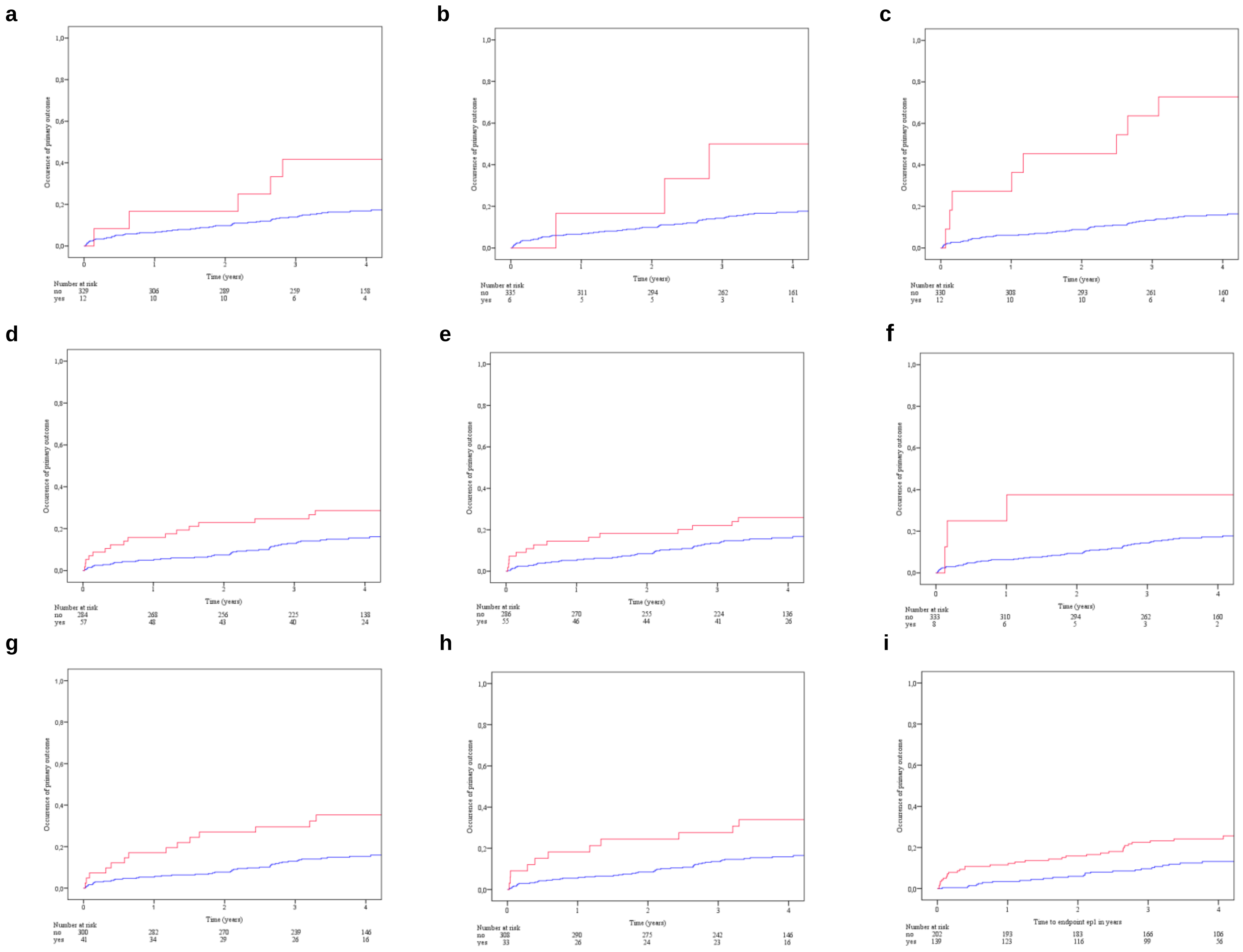
a
Occurrence of primary outcome
1,0
0,8
0,6
0,4
0,2
0,0
0 1 2 3 4
Time (years)
Number at risk
no 329 306 289 259 158
yes 12 10 10 6 4
b
Occurrence of primary outcome
1,0
0,8
0,6
0,4
0,2
0,0
0 1 2 3 4
Time (years)
Number at risk
no 335 311 294 262 161
yes 6 5 5 3 1
c
Occurrence of primary outcome
1,0
0,8
0,6
0,4
0,2
0,0
0 1 2 3 4
Time (years)
Number at risk
no 330 308 293 261 160
yes 12 10 10 6 4
d
Occurrence of primary outcome
1,0
0,8
0,6
0,4
0,2
0,0
0 1 2 3 4
Time (years)
Number at risk
no 284 268 256 225 138
yes 57 48 43 40 24
e
Occurrence of primary outcome
1,0
0,8
0,6
0,4
0,2
0,0
0 1 2 3 4
Time (years)
Number at risk
no 286 270 255 224 136
yes 55 46 44 41 26
f
Occurrence of primary outcome
1,0
0,8
0,6
0,4
0,2
0,0
0 1 2 3 4
Time (years)
Number at risk
no 333 310 294 262 160
yes 8 6 5 3 2
g
Occurrence of primary outcome
1,0
0,8
0,6
0,4
0,2
0,0
0 1 2 3 4
Time (years)
Number at risk
no 300 282 270 239 146
yes 41 34 29 26 16
h
Occurrence of primary outcome
1,0
0,8
0,6
0,4
0,2
0,0
0 1 2 3 4
Time (years)
Number at risk
no 308 290 275 242 146
yes 33 26 24 23 16
i
Occurrence of primary outcome
1,0
0,8
0,6
0,4
0,2
0,0
0 1 2 3 4
Time to endpoint ep1 in years
Number at risk
no 202 193 183 166 106
yes 139 123 116 99 56

SUPPLEMENTARY FIG. 1 Kaplan-Meier cumulative probability of incidence of primary outcome (composite of vascular death, stroke, myocardial infarction, or retinal infarction) during follow-up

(a) Completely colored visual field, (b) completely black visual field with color, (c) involvement of peripheral visual field only, (d) onset of curtain to above or below, (e) resolution of curtain to above or below, (f) constricting onset of visual field loss, (g) downward onset of loss of vision, (h) upward resolution of loss of vision, (i) occurrence of more than three attacks.

Blue line: history characteristic absent. Red line: history characteristic present.
