# Supplementary material for: Transient monocular blindness and the risk of vascular complications according to subtype: a prospective cohort study
Source: J Neurol. 2016 Jun 17;263(9):1771–7. doi: 10.1007/s00415-016-8189-x (PMC5010823; doi:10.1007/s00415-016-8189-x)
Supplement: Supplementary file 2 — Supplementary material 2 (PDF 118 kb) [file 415_2016_8189_MOESM2_ESM.pdf]

ELECTRONIC SUPPLEMENTARY MATERIAL

Article title:

Transient monocular blindness and the risk of vascular complications according to subtype: a prospective cohort study

Journal name:

Journal of Neurology

Authors:

Eline J. Volkers MD, Richard C.J.M. Donders MD, Peter J. Koudstaal MD, Jan van Gijn MD, Ale Algra MD, L. Jaap Kappelle MD

Address for correspondence:

E.J. Volkers, Julius Center for Health Sciences and Primary Care, University Medical Center Utrecht

Internal mail no. Str. 6.131, P.O. Box 85500, 3508 GA Utrecht, The Netherlands

Phone: +31.88.7569626

Fax: +31.88.7568099

Email: E.J.Volkers@umcutrecht.nl

SUPPLEMENTARY TABLE 1 History characteristics distinguished in all patients

Visual field involved	Negative symptoms	Positive symptoms	Mode of onset/resolution	Speed of onset/resolution	Number of attacks	Duration of symptoms
Complete	Blurred vision	Scintillations	From/to above or below	One second	Only one	<1 minute
Upper or lower half	No vision	Bright field	From/to left or right	Seconds	2 to 3	1–10 minutes
Central	Completely black	Flashing lights	From/to both sides	1–5 minutes	More than 3	10–30 minutes
Peripheral	Completely grey	Jagged lines	Diagonal	5–10 minutes	Unknown	30 minutes–24 hours
Patchy	Completely white	Streaks and shimmer	Diffuse/sudden	Unknown		Unknown
Left or right half	Any combination	Figures	Constricting			
Three quarters	No negative symptoms	Vibration, as with heat	Widening			
One quadrant	Unknown	Completely colored	Unknown			
Upper and lower half, with central sparing		No positive symptoms				
Unknown		Unknown				
